# Spatial Relation Between White Matter Hyperintensities and Incident Lacunes of Presumed Vascular Origin: A 14-Year Follow-Up Study

**DOI:** 10.1161/STROKEAHA.122.039903

**Published:** 2022-10-03

**Authors:** Fang Yi, Mengfei Cai, Mina A. Jacob, José Marques, David G. Norris, Marco Duering, Anil M. Tuladhar, Frank-Erik de Leeuw

**Affiliations:** Department of Geriatrics, Xiangya Hospital, Central South University; National Clinical Research Centre for Geriatric Disorders, Changsha, Hunan, China (F.Y.).; Department of Neurology, Donders Center for Medical Neurosciences, Radboud University Medical Center, Nijmegen, the Netherlands (F.Y., M.C., M.A.J., A.M.T., F.-E.d.L.).; Department of Neurology, Guangdong Neuroscience Institute, Guangdong Provincial People’s Hospital, Guangdong Academy of Medical Sciences, Guangzhou, China (M.C.).; Center for Cognitive Neuroimaging, Donders Institute for Brain, Cognition and Behavior, Nijmegen, the Netherlands (J.M., D.G.N.).; Medical Image Analysis Center (MIAC AG) and qbig, Department of Biomedical Engineering, University of Basel, Switzerland (M.D.).

**Keywords:** incident lacunes, magnetic resonance imaging, small vessel disease, spatial distribution, white matter hyperintensities

## Abstract

The underlying mechanisms of incident lacunes regarding their spatial distribution remain largely unknown. We investigated the spatial distribution pattern and MRI predictors of incident lacunes in relation to white matter hyperintensity (WMH) over 14 years follow-up in sporadic small vessel disease.

**Methods:**

Five hundred three participants from the ongoing prospective single-center Radboud University Nijmegen Diffusion Tensor and Magnetic resonance Cohort (RUN DMC) were recruited with baseline assessment in 2006 and follow ups in 2011, 2015, and 2020. Three hundred eighty-two participants who underwent at least 2 available brain MRI scans were included. Incident lacunes were systematically identified, and the spatial relationship between incident lacunes located in subcortical white matter and WMH were determined using a visual rating scale. Adjusted multiple logistic regression and linear mixed-effect regression models were used to assess the association between baseline small vessel disease markers, WMH progression, and incident lacunes. Participants with atrial fibrillation were excluded in multivariable analysis.

**Results:**

Eighty incident lacunes were identified in 43 patients (mean age 66.5±8.2 years, 37.2% women) during a mean follow-up time of 11.2±3.3 years (incidence rate 10.0/1000 person-year). Sixty percent of incident lacunes were in the white matter, of which 48.9% showed no contact with preexisting WMH. Baseline WMH volume (odds ratio=2.5 [95% CI, 1.6–4.2]) predicted incident lacunes after adjustment for age, sex, and vascular risk factors. WMH progression was associated with incident lacunes independent of age, sex, baseline WMH volume, and vascular risk factors (odds ratio, 3.2 [95% CI, 1.5–6.9]). Baseline WMH volume and progression rate were higher in participants with incident lacunes in contact with preexisting WMH. No difference in vascular risk factors was observed regarding location or relation with preexisting WMH.

**Conclusions:**

The 2 different distribution patterns of lacunes regarding their relation to WMH may suggest distinct underlying mechanisms, one of which may be more closely linked to a similar pathophysiology as that of WMH. The longitudinal relation between WMH and lacunes further supports plausible shared mechanisms between the 2 key markers.

Cerebral small vessel disease (SVD) is the most important vascular contributor of cognitive impairment, dementia and causes up to 25% of ischemic strokes.^1^ It is frequently seen on neuroimaging of elderly, for example, white matter hyperintensities (WMHs) and lacunes of presumed vascular origin.^2^ Knowledge in the evolution of SVD such as incident lacunes can help to better understand the origin and consequences of SVD. However, the underlying mechanisms causing incident lacunes remain largely unknown. This is because incident lacunes are difficult to capture as they are typically asymptomatic in the context of sporadic SVD and have a very low incidence,^3,4^ thereby requiring large prospective magnetic resonance imaging (MRI) studies with a long follow-up.

The anatomical location of incident lacunes may provide additional information regarding the underlying mechanisms. For example, basal ganglia lacunes more often have a proximal embolic source (eg, carotid stenosis, atrial fibrillation) than centrum semiovale lesions,^5^ whereas hypoperfusion may be involved in incident lacunes located in centrum semiovale.^6^ Meanwhile, a study in patients with cerebral autosomal dominant arteriopathy with subcortical infarcts and leukoencephalopathy (CADASIL) found that >90% of incident lacunes develop at the border of a WMH,^7^ suggesting a link in the progression of WMH and lacunes. However, external validity is limited as this study was conducted in monogenic SVD. The authors attempted to confirm their findings in patients with sporadic SVD, but due to paucity of incident lacunes, this relation was only observed for prevalent lacunes.^7^ Whether this can be generalized to incident lacunes is unknown. The low proportion of incident lacunes occurring within preexisting WMH indicated that only very few areas within a WMH changed/converted into cavities, which was hypothesized to be one of the mechanisms of subcortical white matter lacunes.^6^ This raises an interest of different etiology of lacunes in terms of their spatial relation to WMH that has not been investigated to date.

We therefore aimed to assess the spatial distribution pattern of incident lacunes and their association with WMH in a large cohort of sporadic SVD with 14-year follow-up. First, we described the characteristics of incident lacunes, stratified by their anatomical location and spatial relationship with preexisting WMH. Second, we investigated the association between baseline SVD imaging markers, WMH progression, and incident lacunes.

## Methods

### Study Population

This study is embedded in the RUN DMC study (Radboud University Nijmegen Diffusion Tensor and Magnetic Resonance Cohort)‚ a prospective single-center study that aims to investigate the risk factors and clinical consequences of sporadic SVD. The detailed study protocol has been published previously.^8^ Main inclusion criteria were (1) age between 50 and 85 years; (2) SVD on neuroimaging, for example, WMH or presence of lacunes. Main exclusion criteria at baseline included: presence of dementia, parkinsonism, non SVD-related white matter lesions (eg, multiple sclerosis), and life expectancy of <6 months. Baseline data collection was performed in 2006 (baseline), with 3 follow-ups (follow-up 1 in 2011, follow-up 2 in 2015, follow-up 3 in 2020). In total, 382 participants who underwent at least 2 available MRI assessments were included in the current study (Figure S1). This study was approved by The Medical Review Ethics Committee region Arnhem-Nijmegen, the Netherlands. All participants signed informed consent. The data that support the findings of this study are available from the corresponding author upon reasonable request. This article follows the STROBE reporting guideline (with reference).

### Vascular Risk Factors

We assessed body mass index, presence of hypertension, smoking, atrial fibrillation, diabetes, hypercholesterolemia, orthostatic hypotension, systolic and diastolic blood pressures at baseline, and follow-ups by standardized assessment and questionnaires, as described previously.^8^ Systolic and diastolic blood pressures were measured 3 times (separated by 1–2 minutes) in supine position after 5 minutes of rest during each visit. Hypertension was defined as SBP≥140 mmHg and/or DBP≥90 mmHg and/or the use of blood pressure–lowering agents. Diabetes and hypercholesterolemia were present if the participant was taking oral glucose-lowering drugs or insulin or lipid-lowering drugs, respectively. Orthostatic hypotension was defined as either a lowering of 20 mmHg of systolic blood pressure or 10 mmHg of diastolic blood pressure after 1 minute of standing up.^9^

### MRI Protocol

Images were acquired at baseline and follow-up 1, 2 on 1.5-Tesla MRI (2006: Siemens, Magnetom Sonata; 2011 and 2015: Siemens, Magnetom Avanto). The same 8-channel head coil was used at all 3 time points. MRI scans at follow-up 3 (2020) were performed on a 3-Tesla MRI scanner (Siemens, Magnetom Prisma) with a 32-channel head coil. Detailed MRI acquisition parameters for each follow-up were shown in Table S1.

### MRI Markers of SVD and Brain Volumetry

WMH, lacunes, and microbleeds were rated in accordance with the STRIVE criteria (Standards for Reporting Vascular Changes on Neuroimaging).^2^ Lacunes were carefully differentiated from enlarged perivascular spaces (see below). WMH at baseline and follow-ups were segmented semiautomatically using FLAIR and T1 sequences as described previously.^10^ To minimize the effects of changes in FLAIR acquisition sequence parameters, we resliced follow-up FLAIR images to match slice thickness of baseline images using FMRIB’s Linear Image Registration Tool (FLIRT), part of FSL.

Gray matter (GM), white matter (WM), and cerebrospinal fluid probability maps were produced using a SPM12 (http://www.fil.ion.ucl.ac.uk/spm/) tissue segmentation algorithm on the acquired T1-weighted images. GM, WM, and cerebrospinal fluid volumes were computed by summing all voxels belonging to that tissue class multiplied by voxel volume in ml. Total brain volume was calculated by summing GM and WM volumes. Intracranial cavity volume was determined by taking the sum of GM, WM, and cerebrospinal fluid volumes. WMH volumes were corrected to baseline intracranial cavity volume.

### Identification of Incident Lacunes

To accurately identify incident lacunes, difference maps were created (Figure S2).^11^ The follow-up images from 2011, 2015, and 2020 were registered to the baseline scans from 2006. After an intensity normalization step,^12^ the difference maps were subsequently acquired by subtracting the T1 and FLAIR follow-up images from the respective baseline images, thus offering a detailed visualization of the changes between multiple images over time. Incident lacunes were independently rated by 2 researchers blinded for clinical data on difference images from 3D T1 and FLAIR followed by a consensus meeting in case of disagreement. Intra-rater and interrater reliabilities were excellent with weighted κ values of 0.92 and 0.89, respectively.^13^ Incident lacunes were rated by location (subcortical areas of the frontal, parietal, temporal, and occipital lobes, centrum semiovale, corona radiata, and corpus callosum were defined as subcortical WM; internal capsule, thalamus and basal ganglia were defined as basal ganglia; brainstem and cerebellum were defined as infratentorial area).

### Rating of Spatial Relationship Between Incident Lacunes and WMH

**Figure 1.**
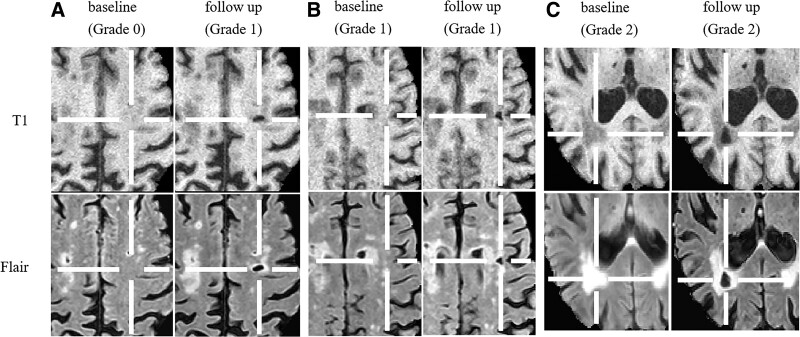
**Rating examples.** Baseline: the last scan before the appearance of each incident lacune. Follow-up: the scan on which each incident lacune occurred. **A**, The incident lacune in left centrum semiovale (center of cross) occurred in normal appearing white matter (no contact with preexisting white matter hyperintensity [WMH]) at baseline and connected with WMH at follow-up. **B**, The incident lacune (center of cross) appeared in brain region showing partial overlap with preexisting WMH. **C**, An incident lacune (center of cross) appeared within a preexisting WMH.

Visual rating scale^7^ was used to assess the spatial relationship between incident lacunes and WMH from 3D FLAIR scans. Because there was hardly any WMH in deep GM, only incident lacunes located in subcortical WM were involved in the assessment. We investigated both the scan on which each incident lacune occurred and the last scan before its appearance. Three categories were used to rate the spatial relation between incident lacunes and WMH: grade 0: no contact between the incident lacune and (preexisting) WMH, grade 1: connection or partial overlap and grade 2: complete overlap/incident lacune occurred within a (preexisting) WMH (Figure 1). Small rims around the incident lacunes on FLAIR scans were not considered as WMH.

### Statistical Analysis

Characteristics of study population were presented as mean±SD for normally distributed data, median and interquartile ranges (IQR) for the skewed distributed parameters. For group comparisons, we used χ^2^ test for dichotomous variables, student *t* test for continuous variables and Wilcoxon Mann-Whitney *U*-test for non-normally distributed data, when appropriate. Lacunes and microbleeds at baseline were dichotomized as present or absent.

Multiple logistic regression models were applied to identify the relation between baseline MRI measures, WMH progression and incident lacunes. Theoretically, to minimize the probability of embolic sources, participants with either large artery disease or cardiac disease (eg, atrial fibrillation) should be excluded. Due to the lack of data on carotid artery ultrasonography and intracranial angiography in our cohort, we only excluded participants with atrial fibrillation. We calculated odds ratios and 95% CIs for each MRI measurement at baseline (ie, WM volume, GM volume, TB volume, WMH volume, presence of lacunes, and microbleeds). Variables with a significance level of *P*≤0.10 in univariate analysis were put into adjusted multiple logistic regression models; one was adjusted for age and sex, and the other was additionally for vascular risk factors (ie, smoking, diabetes, hypercholesterolemia, hypertension, systolic blood pressure, diastolic blood pressure, body mass index, orthostatic hypotension). WMH volume was log-transformed to adjust for its skewed distribution.

To investigate the relation between WMH progression and incident lacunes, we used WMH progression rate over 14 years as an independent variable adjusting for age, sex, baseline WMH volume, and additionally for vascular risk factors. WMH progression rate was estimated using linear mixed-effect models, with random effects of intercept and slope (with respect to follow-up time in year). The fixed effect of time represents the average annualized change of WMH across the whole cohort, while random effects of intercept and slope per participant can allow for inter-individual variability.

All statistical analyses were carried out in R, version 4.1.1 (https:www.r-project.org/). Two-tailed *P*<0.05 were considered statistically significant.

## Results

### Main Characteristics of Incident Lacunes in the Total Study Population

**Figure 2.**
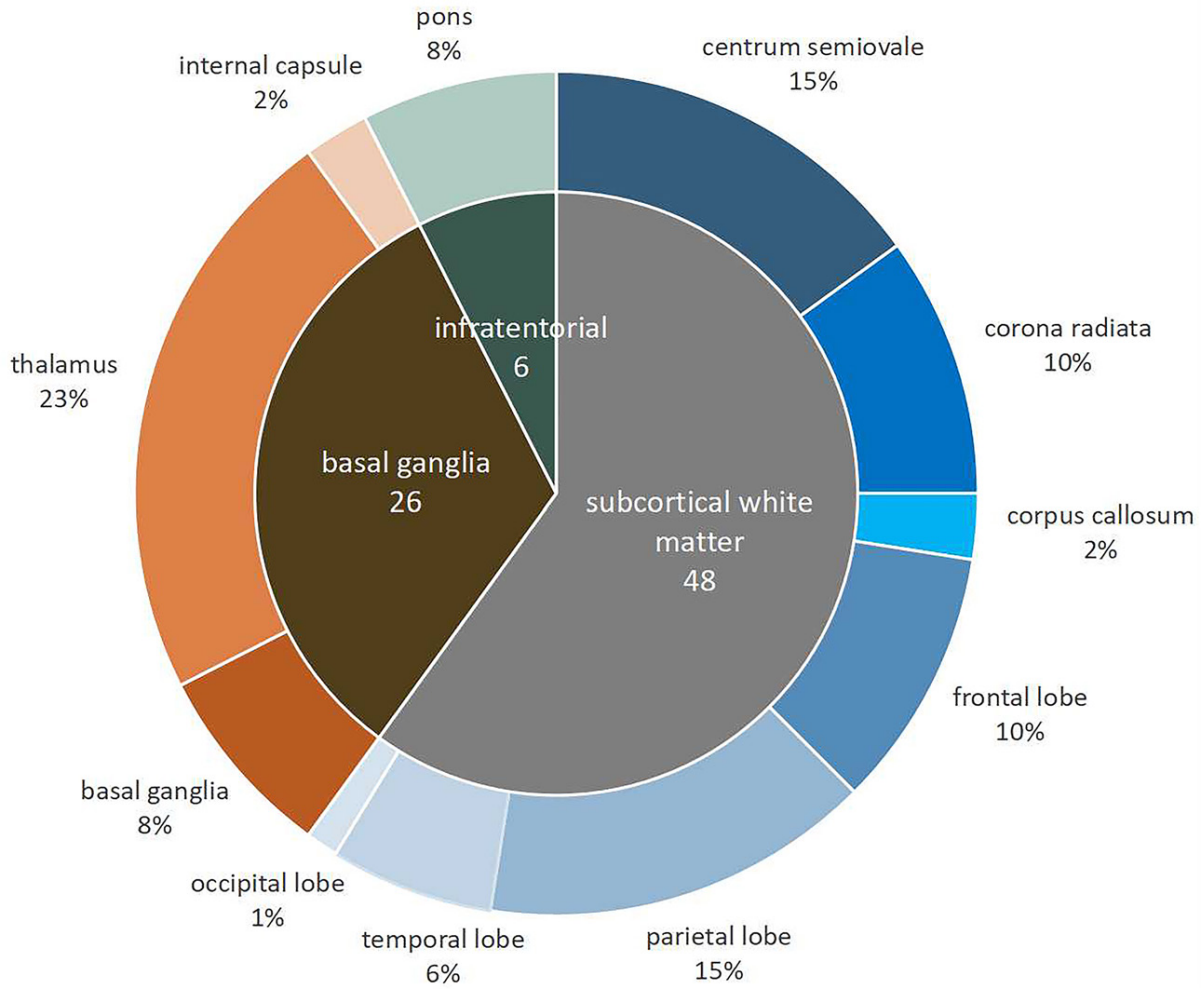
**Details in spatial distribution of incident lacunes.** This Figure shows the proportion of incident lacunes in brain regions. Forty eight incident lacunes were detected in the subcortical white matter, 26 in basal ganglia‚ and 6 in pons.

Baseline characteristics of the total study population are presented in Table 1. Eighty incident lacunes were identified in 43 participants (mean age, 66.5 [SD 8.2], 37.2% women) during a mean follow-up time of 11.2 (SD 3.3) years (incidence rate of 10 per 1000 person-year). Forty-eight incident lacunes (60%) were detected in the subcortical WM. Twenty-six incident lacunes were in the basal ganglia, and 6 infratentorial (all in pons) (Figure 2). Among patients with incident lacunes, 30 (69.8%) had 1, 9 (20.9%) had 2, and 4 patients (9.3%) had >2 incident lacunes.

**Table 1.**
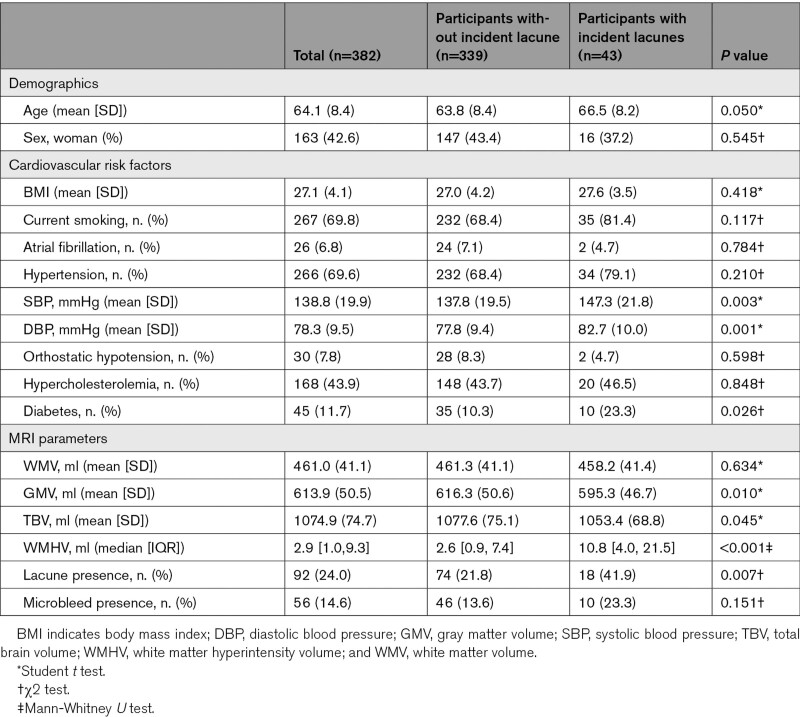
Characteristics of Study Population

### Spatial Relation Between WMH and Incident Lacunes

**Figure 3.**
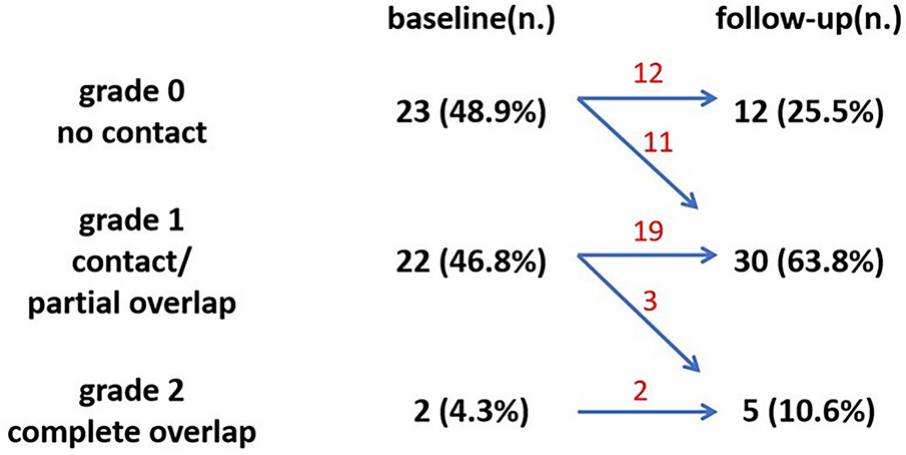
**Spatial relationship between incident white matter lacunes and white matter hyperintensities.** Twenty-three of the 47 incident lacunes detected in the subcortical white matter were rated grade 0 (48.9%), 22 were rated grade 1 (46.8%), and 2 were grade 2 (4.3%) at baseline. Eleven out of 23 lesions (47.8%) rated as grade 0 were presenting grade 1 on follow-up scans. Three out of 22 lesions (13.6%) rated as grade 1 at baseline became fully inside WMH on follow-up scans.

Two incident lacunes detected from 2 participants with atrial fibrillation were excluded, with the location respectively in the thalamus and parietal subcortical WM. Of the other 47 incident subcortical WM lacunes, 23 (48.9%) developed in isolation from a preexisting WMH, 22 (46.8%) developed at the border, whereas 2 (4.3%) occurred within a preexisting WMH (Figure 3). Eleven out of the 23 lacunes (47.8%) rated as grade 0 at baseline showed a partial overlap with WMH on follow-up scans. Three out of the 22 incident lacunes (13.6%) rated as grade 1 at baseline were fully incorporated inside WMH on follow-up scans.

### Risk Factors of Incident Lacunes in Participants Regarding Spatial Distribution

Participants with atrial fibrillation (n=2) were also excluded when investigating risk factors of incident lacunes regarding spatial distribution. To avoid overlapping and consequently possible confounding, baseline risk factors and MRI measures were compared between participants with incident lacunes in basal ganglia/brainstem only (n=16) versus subcortical WM only (n=17) and participants with incident subcortical WM lacunes showing contact/complete overlap with preexisting WMH (n=5) versus in isolation from preexisting WMH (n=12; Table S2). The prevalence of vascular risk factors did not differ between these subgroups.

### Baseline MRI Measures and WMH Progression Associated With Incident Lacunes

Multivariable regression models were conducted in participants without atrial fibrillation (n=380). Baseline WMH volume (OR, 2.5 [95% CI, 1.6–4.2]; *P*<0.001) predicted incident lacunes independent of age, sex, and vascular risk factors (Table 2). WMH progression was associated with incident lacunes after adjustment for age, sex, and baseline WMH volume (OR, 3.0 [95% CI, 1.5–6.4]; *P*=0.003), and remained significant additionally adjusting for vascular risk factors (OR, 3.2 [95% CI, 1.5–6.9]). Of note, participants with incident subcortical WM lacunes showing contact/complete overlap with pre-existing WMH had nearly 5-fold higher baseline WMH volume and more than double the WMH progression rate than those without connection (*P*=0.003 and 0.006, respectively), whereas baseline WMH volume and WMH progression rate were similar in participants with incident lacunes in basal ganglia/brainstem only and subcortical WM lacunes only (Table S2).

**Table 2.**
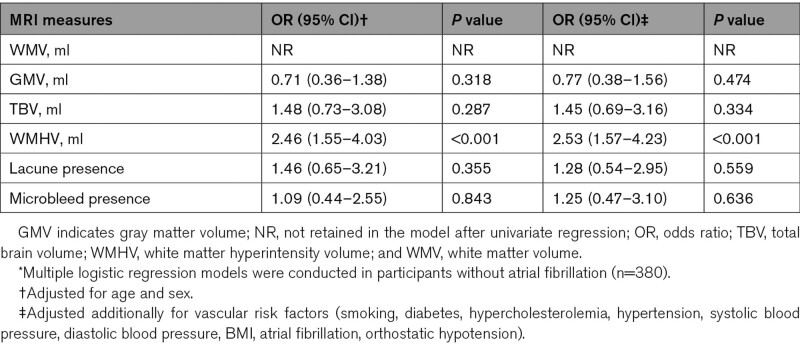
Association Between Baseline MRI Measures and Incident Lacunes*

## Discussion

We found that in individuals with sporadic SVD most incident lacunes (60%) emerged in the subcortical WM, and roughly half (48.9%) developed in isolation from a preexisting WMH. Baseline WMH volume and WMH progression were independently associated with incident lacunes. In addition, baseline WMH volume and WMH progression rate were higher especially in participants with incident lacunes in contact with preexisting WMH. No difference in vascular risk factors was observed regarding location or relation of incident lacunes with preexisting WMH.

This work extends our knowledge in underlying mechanisms of incident lacunes in relation to their spatial distribution. Interestingly, roughly half of incident lacunes in our cohort with sporadic SVD patients were not in contact with preexisting WMH, and still a quarter of them remained in isolation from WMH at follow-up. In those incident lacunes in contact with preexisting WMH, we observed a nearly 5-fold higher baseline WMH volume and more than double the WMH progression rate. The distinct distribution patterns of lacunes may imply that different underlying mechanisms exist regarding their spatial relation to WMH, as those in contact with WMH may more likely have a similar pathophysiology as WMH. The low proportion of incident lacunes that occurred fully within WMH both in our cohort and CADASIL patients^7^ indicates that the cavitation of WMH is an uncommon mechanism of incident lacunes.

Our results were to some extent inconsistent with the previous study conducted in CADASIL patients, reporting a higher number (>90%) of incident lacunes occurring at the border of WMH.^7^ This discrepancy may be in part explained by different etiology of incident lacunes between sporadic SVD and hereditary SVD (ie, CADASIL). CADASIL is genetically defined^14^ and thus pure SVD, affecting also younger patients, who rarely have confounding stroke etiologies. In contrast to CADASIL patients, an embolic etiology of incident lacunes, either in the context of large artery disease or cardiac embolism, cannot be excluded in sporadic SVD patients. To minimize the probability of cardioembolism, we excluded participants with atrial fibrillation. We acknowledge that lack of data on carotid artery ultrasonography and intracranial angiography in our cohort made it difficult to exclude patients with large artery disease. However, the risk of misclassifying large artery disease as SVD seems small owing to the low prevalence of carotid or intracranial stenosis in asymptomatic individuals from Caucasia descent.^15^ Since it was reported that only 10% to 15% of lacunar infarcts are attributed to emboli,^16^ other explanations could also be made for the spatial discrepancy of incident lacunes. First, the possibility of the development of an “in-between follow-ups” WMH in the brain area prior to the emergence of an incident lacune could not be neglected due to the relatively long follow-up interval in our study. Second, the WMH burden is often higher in CADASIL than in sporadic SVD, which may make it more likely for a lacune to occur in connection to a WMH. However, according to the simulated data in the previous study in CADASIL patients, the observed distribution of incident lacunes in connection with WMH was much higher than the calculated distribution, indicating that the WMH burden is not the main reason of the close spatial relationship with incident lacunes.^7^

We found that baseline WMH volume predicted incident lacunes, which is consistent with several, but not all previous studies.^17–20^ In addition, we showed that WMH progression was associated with incident lacunes independent of age, sex, baseline WMH volume, and vascular risk factors. Our results highlight a longitudinal relation between WMH and lacunes. Traditionally, distinct mechanisms are discussed to underly these 2 SVD markers. The majority (80%) of incident lacunes was reported due to acute ischemia lesions,^21^ while most WMH progression would be explained by other mechanisms, for example, demyelination, blood-brain barrier dysfunction, and inflammation.^22,23^ Recently, shared mechanisms between WMH and lacunes have been suggested, which could be supported by a range of shared vascular risk factors,^24,25^ plasma inflammatory markers,^26^ and advancing MRI studies observing a blood-brain barrier dysfunction both in white matter remote from any acute infarct and WMH.^27,28^ Moreover, we found that baseline WMH burden and WMH progression were more severe in participants with incident lacunes in close relation to preexisting WMH than those without connection, underlining plausible shared mechanisms between WMH and such kind of incident lacunes. This suggests, although needs more validation, the subtype of lacunes in connection with WMH may be more likely driven by SVD, rather than embolic sources. Nevertheless, we did not find any difference in vascular risk factors of incident lacunes concerning spatial distribution. This may partly be explained by the relatively younger participants and mild extent of SVD in the current study, therefore resulting in a low incidence of new lacunes and subsequently decreased statistical power. In addition, the temporal dynamics of vascular risk factors during the long follow-ups could affect the presence of incident lacunes. Ideally, the number of incident lacunes should be considered, as it may also related to the potential pathophysiological mechanism. We only observed 2 outliers with >3 incident lacunes in the current study, which made it statistically difficult to treat them as a separate group.

Strengths of this study are the large sample size and long follow-up of 14 years, which enable us to study progression of SVD over a long period. Furthermore, we had a strict criterion in incident lacunes identification to ensure the reliability of the results. Nevertheless, some limitations need to be addressed. First, scanner changes and differences in the acquisition protocol (eg, differences in resolution and field of strength) during the follow-ups could lead to measurement variability in detection of incident lacunes. However, these improvements are almost inevitable for very long-term studies. Second, attrition bias might have led to an underestimate of incidence rate of incident lacunes, as the participants lost to follow-up or unable to complete the entire follow-up had more severe SVD at baseline. Third, differentiating lacunes from perivascular spaces still remains challenging and time-consuming although misclassification has been cautiously avoided.

In conclusion, we found 2 distinct distribution patterns of incident lacunes in our cohort; an associated etiology with WMH may play a major role in those with close spatial relation to WMH, while those in isolation with WMH may mainly be due to other mechanisms, for example, embolism. Future follow-up studies with more detailed assessment of vascular risk factors and changes over time, and with more advanced MRI techniques (eg, arterial spin labeling, diffusion weighted imaging) would be needed to further illustrate the pathophysiological mechanisms of incident lacunes.

## Article Information

### Acknowledgments

We thank all participants in this study for their valuable contributions.

### Sources of Funding

This study was supported by a VIDI innovational grant 016.126.351 from The Netherlands Organization for Scientific Research (Dr. de Leeuw). AMT is a junior staff member of the Dutch Heart Foundation (grant number 2016T044). Drs Yi and Cai are supported by China Scholarship Council (201906375014 and 201706100189, respectively). Furthermore, this study was supported by the Neurology Research Pool, University Hospital Basel.

### Disclosures

None.

### Supplemental Material

STROBE checklist

Tables S1–S2

Figures S1–S2

## Supplementary Material


