# Supplementary material for: Spatial Relation Between White Matter Hyperintensities and Incident Lacunes of Presumed Vascular Origin: A 14-Year Follow-Up Study
Source: Stroke. 2022 Oct 3;53(12):3688–95. doi: 10.1161/STROKEAHA.122.039903 (PMC9698104; doi:10.1161/STROKEAHA.122.039903)
Supplement: Supplementary file 1 [file str-53-3688-s001.pdf]

SUPPLEMENTAL MATERIAL

STROBE Statement—Checklist of items that should be included in reports of ***cohort studies***

	Item No	Recommendation	Page No.
**Title and abstract**	1	(*a*) Indicate the study's design with a commonly used term in the title or the abstract	1,4
		(*b*) Provide in the abstract an informative and balanced summary of what was done and what was found	4
**Introduction**			
Background/rationale	2	Explain the scientific background and rationale for the investigation being reported	6
Objectives	3	State specific objectives, including any prespecified hypotheses	6-7
**Methods**			
Study design	4	Present key elements of study design early in the paper	7
Setting	5	Describe the setting, locations, and relevant dates, including periods of recruitment, exposure, follow-up, and data collection	7
Participants	6	(*a*) Give the eligibility criteria, and the sources and methods of selection of participants. Describe methods of follow-up	7
		(*b*) For matched studies, give matching criteria and number of exposed and unexposed	7
Variables	7	Clearly define all outcomes, exposures, predictors, potential confounders, and effect modifiers. Give diagnostic criteria, if applicable	7-9
Data sources/ measurement	8*	For each variable of interest, give sources of data and details of methods of assessment (measurement). Describe comparability of assessment methods if there is more than one group	7-9
Bias	9	Describe any efforts to address potential sources of bias	9-10,14
Study size	10	Explain how the study size was arrived at	7,11
Quantitative variables	11	Explain how quantitative variables were handled in the analyses. If applicable, describe which groupings were chosen and why	9-10
Statistical methods	12	(*a*) Describe all statistical methods, including those used to control for confounding	9-10
		(*b*) Describe any methods used to examine subgroups and interactions	9-11
		(*c*) Explain how missing data were addressed	NA
		(*d*) If applicable, explain how loss to follow-up was addressed	10
		(*e*) Describe any sensitivity analyses	NA
**Results**			

Participants	13*	(a) Report numbers of individuals at each stage of study—eg numbers potentially eligible, examined for eligibility, confirmed eligible, included in the study, completing follow-up, and analysed	7,10-11
		(b) Give reasons for non-participation at each stage	11
		(c) Consider use of a flow diagram	Figure S1
Descriptive data	14*	(a) Give characteristics of study participants (eg demographic, clinical, social) and information on exposures and potential confounders	Table 1
		(b) Indicate number of participants with missing data for each variable of interest	NA
		(c) Summarise follow-up time (eg, average and total amount)	10
Outcome data	15*	Report numbers of outcome events or summary measures over time	10-11
Main results	16	(*a*) Give unadjusted estimates and, if applicable, confounder-adjusted estimates and their precision (eg, 95% confidence interval). Make clear which confounders were adjusted for and why they were included	11, Table 2
		(*b*) Report category boundaries when continuous variables were categorized	NA
		(*c*) If relevant, consider translating estimates of relative risk into absolute risk for a meaningful time period	NA
Other analyses	17	Report other analyses done—eg analyses of subgroups and interactions, and sensitivity analyses	11-12, Table S2
**Discussion**			
Key results	18	Summarise key results with reference to study objectives	12
Limitations	19	Discuss limitations of the study, taking into account sources of potential bias or imprecision. Discuss both direction and magnitude of any potential bias	14
Interpretation	20	Give a cautious overall interpretation of results considering objectives, limitations, multiplicity of analyses, results from similar studies, and other relevant evidence	12-14
Generalisability	21	Discuss the generalisability (external validity) of the study results	13
**Other information**			
Funding	22	Give the source of funding and the role of the funders for the present study and, if applicable, for the original study on which the present article is based	3

*Give information separately for exposed and unexposed groups.

Note: An Explanation and Elaboration article discusses each checklist item and gives methodological background and published examples of transparent reporting. The STROBE checklist is best used in conjunction

with this article (freely available on the Web sites of PLoS Medicine at http://www.plosmedicine.org/, Annals of Internal Medicine at http://www.annals.org/, and Epidemiology at http://www.epidem.com/). Information on the STROBE Initiative is available at http://www.strobe-statement.org.

Table S1: MRI acquisition parameters

Sequence	Feature	2006	2011	2015	2020
	Scanner	Magnetom Sonata 1.5 Tesla	Magnetom Avanto 1.5 Tesla	Magnetom Avanto 1.5 Tesla	Magnetom Prisma 3 Tesla
	Coil Channels	8 (head)	8 (head)	8 (head)	32 (head)
3D-T1	Type	MPRAGE	MPRAGE	MPRAGE	MP2RAGE
	TR (ms)	2250	2250	2250	5500
	TE (ms)	3.68	2.95	2.95	3.84
	TI (ms)	850	850	850	700/2500
	Voxel size [mm]	1.0 isotropic	1.0 isotropic	1.0 isotropic	0.85 isotropic
3D-FLAIR	TR (ms)	9000	14240	14240	5000
	TE (ms)	84	89	89	394
	TI (ms)	2200	2200	2200	180
	Voxel size [mm]	1.2×1.0×5.0	1.2×1.0×2.5	1.2×1.0×2.5	0.85 isotropic

Abbreviations: FLAIR = fluid-attenuated inversion recovery; MP2RAGE = magnetization prepared (2) rapid acquisition gradient echo(es); TE = echo time; TI = inversion time; TR = repetition time

Table S2: Factors in participants by distribution of incident lacunes

	Basal ganglia / brainstem only n=16	Subcortical WM only n=17	Subcortical WM only		
			Grade 0 n=12	Grade 1-2 n=5	P-value§
**Demographics**					
Age (mean (SD))	68.8 (7.8)	64.3 (9.3)	62.2 (8.7)	69.3 (9.7)	0.157*
Sex, woman (%)	7 (43.8)	6 (35.3)	5 (41.7)	1 (20.0)	0.768†
**Cardiovascular risk factors**					
BMI (mean (SD))	27.6 (4.3)	28.5 (2.8)	28.4 (2.9)	28.8 (3.1)	0.808*
Smoking, n. (%)	12 (75.0)	14 (82.4)	9 (75.0)	5 (100.0)	0.593†
Hypertension, n. (%)	13 (81.2)	12 (70.6)	8 (66.7)	4 (80.0)	1.000†
SBP, mmHg (mean (SD))	154.6 (25.8)	140.4 (20.3)	137.9 (20.4)	146.2 (21.1)	0.461*
DBP, mmHg (mean (SD))	85.9 (12.2)	80.7 (9.0)	79.1 (9.2)	84.4 (8.1)	0.279*
Orthostatic hypotension, n. (%)	0 (0.0)	2 (11.8)	2 (16.7)	0 (0.0)	0.884†
Hypercholesterolemia, n. (%)	7 (43.8)	8 (47.1)	6 (50.0)	2 (40.0)	1.000†
Diabetes, n. (%)	2 (12.5)	4 (23.5)	3 (25.0)	1 (20.0)	1.000†
**MRI parameters**					
WMV, ml (mean (SD))	452.2 (49.9)	459.1 (42.6)	458.3 (35.4)	460.9 (61.6)	0.915*
GMV, ml (mean (SD))	589.3 (38.9)	605.2 (56.6)	629.9 (39.2)	545.9 (48.5)	**0.002***
TBV, ml (mean (SD))	1041.5 (73.3)	1064.3 (77.7)	1088.2 (49.4)	1006.8 (107.7)	**0.045***
WMHV, ml (median [IQR])	6.3 [4.2, 16.5]	11.0 [2.8, 24.2]	7.1 [2.2, 12.3]	35.0 [26.3, 40.6]	**0.003**‡
Lacune presence, n. (%)	6 (37.5)	7 (41.2)	4 (33.3)	3 (60.0)	0.633†
Microbleed presence, n. (%)	1 (6.2)	4 (23.5)	2 (16.7)	2 (40.0)	0.685†
WMHV progression rate, ml/y (median [IQR])	0.7 [0.5, 2.4]	0.8 [0.7, 1.6]	0.7 [0.3, 0.9]	1.8 [1.6, 1.8]	**0.006**‡

Abbreviations: BMI: body mass index. SBP, systolic blood pressure; DBP, diastolic blood pressure; WMV, white matter volume; GMV, gray matter volume; TBV, total brain volume; WMHV, white matter hyperintensity volume.

*student t test, †χ2 test, ‡Mann-Whitney U-test

§ Group comparisons between grade 0 and grade1-2.

Figure S1. Flowchart of RUN DMC study population over time

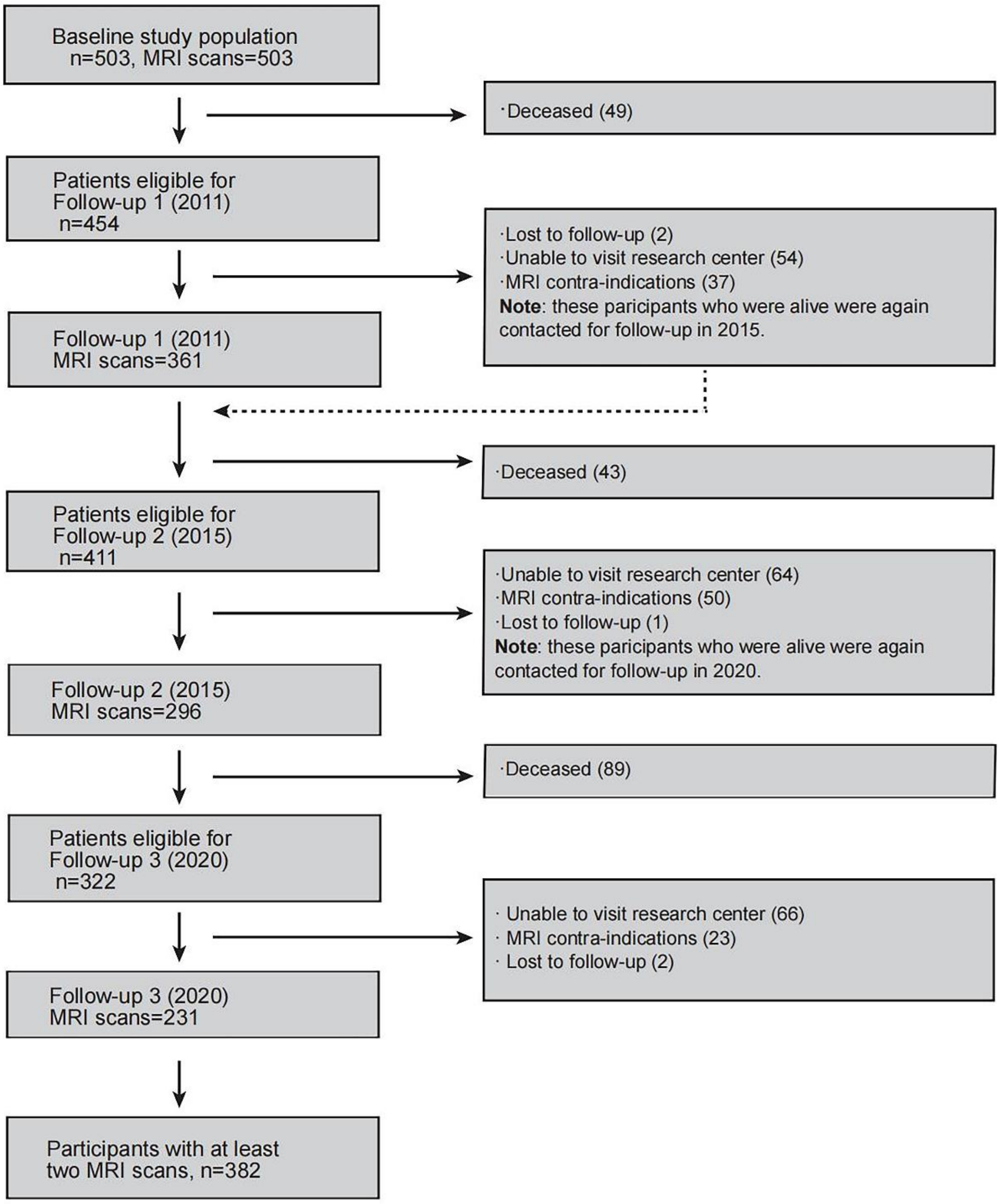

Figure S2. Identification of incident lacunes

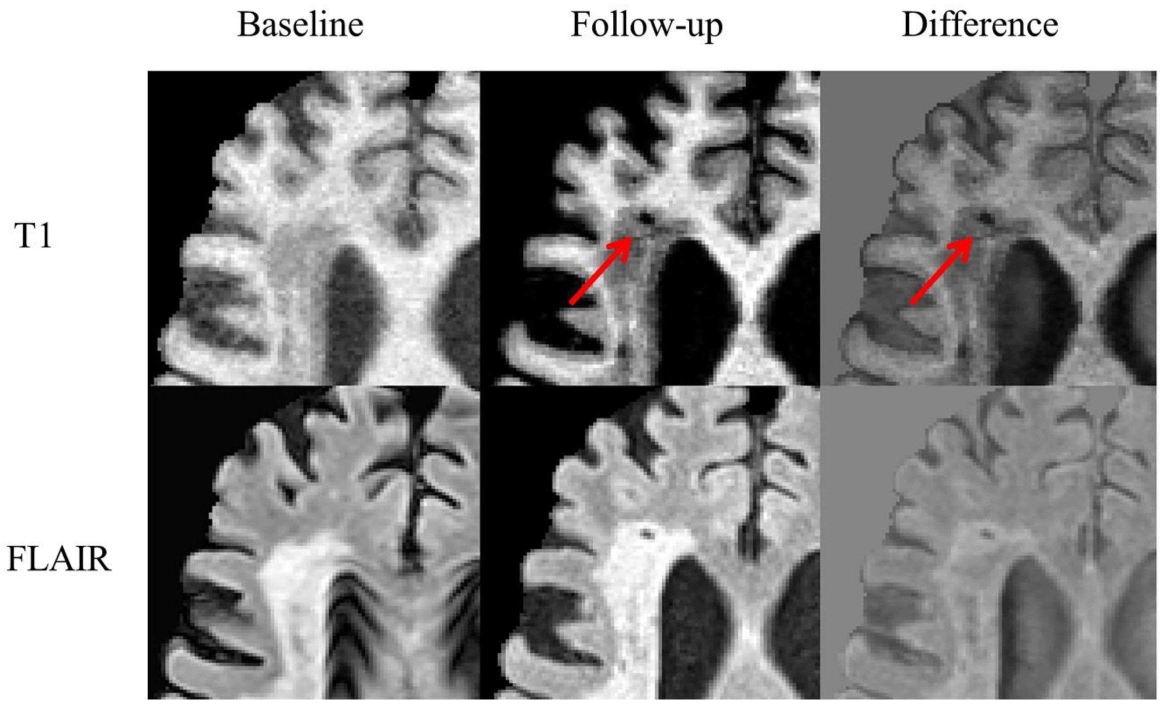

An incident lacune developed in right frontal lobe. Difference maps were acquired by deducting/subtracting the T1-weighted follow-up images from the baseline images.
